# Amphibian and reptile dataset across different land-use types in Guinea-Bissau, West Africa

**DOI:** 10.3897/BDJ.13.e147388

**Published:** 2025-04-24

**Authors:** Francisco dos Reis-Silva, Fernanda Alves-Martins, Javier Martínez-Arribas, Cristian Pizzigalli, Sambu Seck, Ana Rainho, Ricardo Rocha, Ana Filipa Palmeirim

**Affiliations:** 1 Global Change and Conservation Research Group, Faculty of Biological and Environmental Sciences, University of Helsinki, Viikinkaari, Finland Global Change and Conservation Research Group, Faculty of Biological and Environmental Sciences, University of Helsinki Viikinkaari Finland; 2 CIBIO, Centro de Investigação em Biodiversidade e Recursos Genéticos, InBIO Laboratório Associado, Campus de Vairão, Universidade do Porto, Vairão, Portugal CIBIO, Centro de Investigação em Biodiversidade e Recursos Genéticos, InBIO Laboratório Associado, Campus de Vairão, Universidade do Porto Vairão Portugal; 3 BIOPOLIS Program in Genomics, Biodiversity and Land Planning, CIBIO, Campus de Vairão, Vairão, Portugal BIOPOLIS Program in Genomics, Biodiversity and Land Planning, CIBIO, Campus de Vairão Vairão Portugal; 4 Federação KAFO, Guiné-Bissau CP 1186, Centro Camponês de Djalicunda, sector de Mansaba, região de Oio, Djalicunda, Guinea-Bisau Federação KAFO, Guiné-Bissau CP 1186, Centro Camponês de Djalicunda, sector de Mansaba, região de Oio Djalicunda Guinea-Bisau; 5 Centre for Ecology, Evolution and Environmental Changes & CHANGE - Global Change and Sustainability Institute. Departamento de Biologia Animal, Faculdade de Ciências, Universidade de Lisboa, Lisboa, Portugal Centre for Ecology, Evolution and Environmental Changes & CHANGE - Global Change and Sustainability Institute. Departamento de Biologia Animal, Faculdade de Ciências, Universidade de Lisboa Lisboa Portugal; 6 Department of Biology, University of Oxford, 11a Mansfield Rd, OX1 3SZ, Oxford, United Kingdom Department of Biology, University of Oxford, 11a Mansfield Rd, OX1 3SZ Oxford United Kingdom; 7 EcoHealth Alliance, New York City, United States of America EcoHealth Alliance New York City United States of America

**Keywords:** agroecosystems, habitat conversion, herpetofauna, species diversity, tropical forest, Wallacean shortfall, West Africa

## Abstract

**Background:**

West Africa is exceptionally biodiverse, yet its wildlife remains largely understudied despite the rapid and ongoing land-use changes. Large swaths of Guinea-Bissau’s landscape were historically characterised by native forest-savannah mosaics. However, key areas of savannah habitats have been converted to rice agroecosystems and forests are being transformed into cashew monocultures at unprecedented rates. Amphibians and reptiles comprise some of the most threatened species by human-induced habitat change and yet are not as studied as other vertebrate terrestrial taxa. Here, we provide two comprehensive datasets on amphibians and reptiles (classes Testudines and Squamata) from northern Guinea-Bissau: (1) a standardised survey dataset (encompassing sampling events and occurrences) in forest fragments, cashew orchards and rice paddies and (2) an opportunistic dataset reporting occurrences across the entire study area. Standardised surveys were carried across 21 sampling sites, seven in each habitat type, while opportunistic surveys include all other records. For standardised surveys, a total of 703 amphibian and 265 reptile (class Squamata) encounters are reported, corresponding to nine and 13 taxa, respectively. Opportunistically, we report 62 amphibian and 93 reptile encounters, corresponding to 10 amphibian taxa, 25 Squamata taxa and two turtles (class Testudines).

**New information:**

Based on 126 sampling hours of both diurnal and nocturnal standardised surveys, in addition to opportunistic surveys, these datasets comprise the first overview for amphibians and reptiles in mainland Guinea-Bissau across two seasons and different habitat types. Each of the 968 standardised and 155 opportunistic occurrences corresponds to a genus or species and is accompanied by geographic coordinates, a timestamp and, for standardised data, the land-use type. The datasets fill the distribution gaps in Guinea-Bissau of at least three species, including the frog *Hildebrandtiaornata*, the skink *Trachylepiskeroanensis* and the snake *Dendroaspispolylepis* – and include the re-discovery of the lizard *Latastiaornata* in Guinea-Bissau. Before this work, the *L.ornata* was only known from the 1938 holotype in Bafatá (ca. 60 km away from the study area) and, in 2023, from Guinea-Conakry (ca. 700 km away from the type specimen location).

## Introduction

West Africa is a major biodiversity hotspot, with a high number of endemic species (Myers et al. 2000). The region has faced substantial habitat loss and degradation (Lewin et al. 2016), which is expected to continue (Powers and Jetz 2019). Yet, West Africa has been subject to very few ecological studies compared to other biodiversity hotspots, such as the Neotropics (Gardner et al. 2009, Gibson et al. 2011, Newbold et al. 2020, but see Vasconcelos et al. 2015 and Rossinyol-Fernàndez et al. 2024). In fact, a substantial Wallacean shortfall still exists here, reflecting the geographic bias in species distribution data (Hortal et al. 2015).

In West Africa, Guinea-Bissau has been covered by native forest-savannah mosaics (Catarino et al. 2008), but its long history of agriculture has changed the landscape over time (Temudo and Abrantes 2013). Rice (*Oryza* L.) has traditionally been cultivated for domestic use (Temudo and Abrantes 2013) and, together with groundnuts, comprised the core of the agricultural land in the country until the 20^th^ century (Catarino et al. 2015). After the 1940s, cashew trees (*Anacardiumoccidentale* L.) – native to the northeast region of Brazil – started being systematically planted across the country (Temudo and Abrantes 2014). This global agricultural commodity (Rege and Lee 2023) has replaced most other forms of land use in Guinea-Bissau, especially since the 1980s (Temudo and Abrantes 2013). Today, agriculture is still the main source of livelihood in the country, with cashew nuts comprising the only cash crop for the economy of Guinea-Bissau (Temudo and Abrantes 2013), accounting for 90% of all exports (FAO 2021). The once highly complex bio-cultural landscapes in Guinea-Bissau are now threatened by the quick expansion of cashew orchards, which are homogenising the landscape (Catarino et al. 2015, Guedes et al. 2024). To date, little is known about how species diversity copes with land-use change here, which is precluded by the lack of general knowledge on the existing species diversity (Catarino et al. 2015, Guedes et al. 2024).

The lack of information on species diversity in Guinea-Bissau is particularly accute for amphibians and reptiles (Jenkins et al. 2013, Guedes et al. 2023), which are amongst the most threatened vertebrates worldwide (Cox et al. 2022). For instance, the lizard *Latastiaornata* (Monard, 1940) was only known from one type locality specimen for over 80 years (Meiri et al. 2018, Pauwels et al. 2023) or the medically significant black mamba, *Dendroaspispolylepis* (Günther, 1864) that has only a few scattered observations in West Africa when, in fact, its distribution is suspected to be widespread in the region (Chippaux and Jackson 2019). This lack of information, as seen with venomous snakes, also affects human well-being, as it may contribute to a higher incidence of untreated snakebites (WHO 2023). To this day, we only know of one peer-reviewed article assessing herpetofauna in Guinea-Bissau (Auliya et al. 2012), carried out in the Bijagos Archipelago, leaving continental Guinea-Bissau without systematic sampling. Despite the scarcity of scientific studies, amphibians and reptiles are deeply embedded in the West Africa's biocultural heritage (e.g. notwithstanding considerable levels of dislike towards snakes, Bissau-Guinean farmers often perceive snakes as protectors of the village and signs of a good harvest; Chaves et al. (2024)).

To help fill in the knowledge gap in amphibian and reptile distribution in West Africa, we provide two herpetofauna datasets resulting from standardised and opportunistic surveys in the Oio Province, Guinea-Bissau. By doing so, we make available for future ecological studies the data that resulted in Dos Reis‐Silva et al. (2025) collected through standardised surveys. In addition, to maximise species coverage for the study area, we also make available a second dataset of species opportunistically surveyed, that encompass all observations made outside the standardised surveys during the same period. The Oio Region, encompassing a moisaic of forest remnants, cashew orchards and rice paddies, was specifically chosen to detect a wide range of species associated with both open and closed habitats. Furthermore, data from these surveys also provide information about the species that can be found in the expanding cashew orchards. Unconventionally, the surveys were carried out outside protected areas, which contributes further to overcoming the Wallacean Shortfall.

## General description

### Purpose

These two datasets, consisting of standardised surveys supplemented by opportunistic observations, provide the first comprehensive overview of amphibians and reptiles (classes Testudines and Squamata) across different land-use types in the Oio Province, Guinea-Bissau, West Africa.

### Additional information

**Figure 1.**
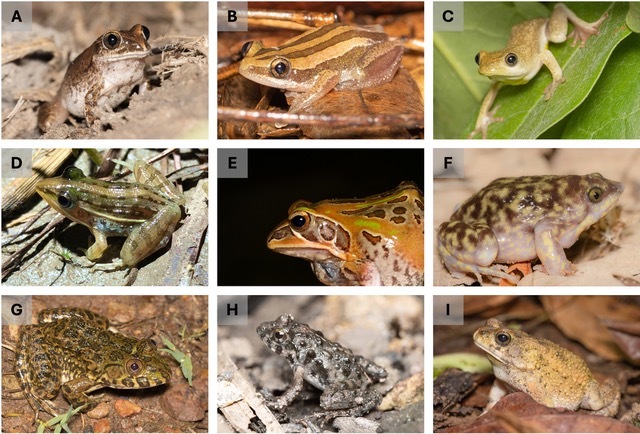
Some of the amphibians observed. **A** *Leptopelisviridis*; **B** *Afrixalusvittiger*; **C** *Hyperoliusspatzi*; **D** *Ptycadena* sp.; **E** *Hildebrandtiaornata*; **F** *Hemisus* sp.; **G** *Hoplobatrachusoccipitalis*; **H** *Phrynobatrachus* sp.; **I** *Slerophrys* sp. Photo credits: Francisco dos Reis-Silva.

**Figure 2.**
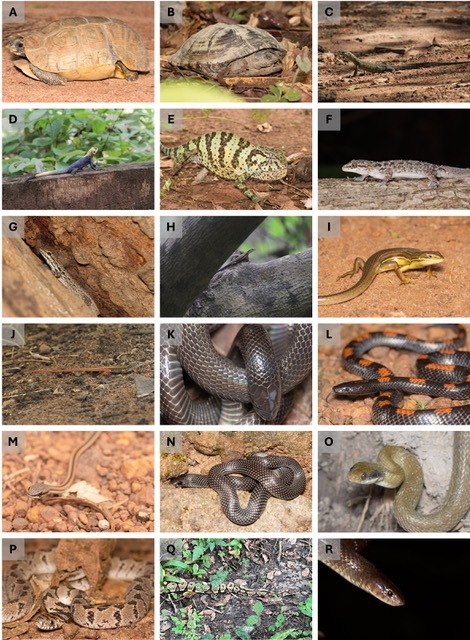
Some of the reptiles observed. **A** *Kinixysbelliana*; **B** *Pelusioscastaneus*; **C** *Varanusniloticus*; **D** *Agamaagama*; **E** *Chamaeleogracillis*; **F** *Hemidactylusangulatus*; **G** *Lygodactylusgutturalis*; **H** *Trachylepisaffinis*; **I** *Trachylepiskeroanensis*; **J** *Latastiaornata*; **K** *Atractaspisaterrima*; **L** *Lycophidionalbomaculatum*; **M** *Psammophiselegans*; **N** *Prosymnameleagris*; **O** *Crotaphopeltishotamboeia*; **P** *Dasypeltisconfusa*; **Q** *Pythonregius*; **R** *Elapsoideasemiannulata*. Photo credits: Francisco dos Reis-Silva (A, B, E, F, G, I, K, L, M, N, O, P), Ricardo Rocha (C, D, H, J) and Cristian Pizzigalli (Q).

The standardised surveys recorded a total of 968 observations, representing nine amphibian and 13 squamate species, respectively. The opportunistic surveys documented 155 records, representing 10 amphibian, 25 squamate and two turtle species. The subset of the amphibian diversity recorded during standardised surveys is shown in Fig. 1 and opportunistic encounters in Fig. 2.

## Sampling methods

### Study extent

**Figure 3.**
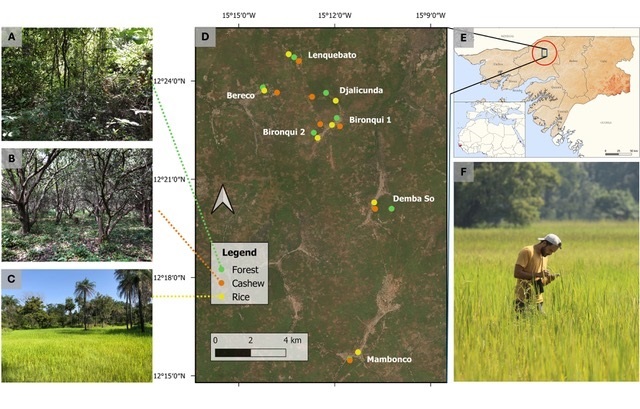
Study area and surveyed habitat types. **A** Forest remnants; **B** Cashew orchards; **C** Rice paddies; **D** Overview of the study area, including study sites (coloured dots corresponding to the habitat type on the legend); **E** Study area in northern Guinea-Bissau; **F** Example of a survey conducted in a rice paddy. Map sources: geoBoundaries (2017) and GADM (2021). Photo credits: Francisco dos Reis-Silva.

This study took place in northern Guinea-Bissau, Oio Province, in the surroundings of Djalicunda (12°19'49"N, 15°10'57"W) (Fig. 3). The area is located in a forest-savannah biome and the landscape surveyed consists of scattered small tabancas (villages) surrounded by secondary forest and areas of small-holder agriculture (Sottomayor et al. 2024). The semi-natural and agricultural areas create mosaics of mostly forest remnants, cashew orchards and rice paddies. Within the region, cashew orchards are gaining prominence, leading to the clearing of some of the forest remnants (Temudo and Abrantes 2014). The area is mostly flat, below 50 m altitude and has been defined as the wet season from June to October and the dry season from October to June (Catarino et al. 2008). The mean temperature in the country ranges between 25.9 and 27.1°C and the annual precipitation is between 1,200 mm in the northeast and 2,600 mm in the southwest (Catarino et al. 2008).

The amphibian and reptile surveys were conducted mainly across three habitat types: forest remnants, cashew orchards and rice paddies. The surveys took place in 21 study sites, seven of each habitat type. Forest remnants in the study area are classified as secondary growth, as they are either heavily degraded or represent re-growth following human intervention (Catarino et al. 2008). In the surveyed forest remnant sites, products (e.g. wood, fruit, honey) are collected by local communities and the ground is typically covered by leaf litter and the canopy cover is ≥ 65% (Dos Reis‐Silva et al. 2025). Surveyed cashew orchard sites are monocultures subject to little management (i.e. no irrigation, no fertilisers). They are characterised by a dense canopy (usually ≥ 80%) about 6–10 m above the ground and the understorey is cleared once a year to facilitate cashew nut harvest (Dos Reis‐Silva et al. 2025). Rice paddies are in topographic depressions that flood naturally between late July and November, which coincides with the plantation and harvesting of rice, respectively (Sottomayor et al. 2024). They have few scattered trees throughout, presenting an open habitat without canopy cover.

### Sampling description

Data collection took place over two field campaigns in 2022. To maximise the number of recorded species given the strong seasonality in the study area, the first field campaign occurred at the end of the dry season (June/July) and the second one at the end of the wet season (October/November). For each campaign, all sampling sites were surveyed three times during the day (starting between 09:15 h and 16:45 h) and once at night (starting between 19:00 h and 22:45 h), totalling eight surveys at each of the 21 sites (six day- and two night-surveys). Details on sampling sufficiency across sampling sites, habitat type and class are presented in Dos Reis‐Silva et al. (2025).

Standardised herpetofauna surveys took place across 21 circular study sites of 25 m radius in a time-standardised fashion (Dos Reis‐Silva et al. 2025). Surveys were systematically conducted by one observer for 45 minutes, amounting to a total of 126 sampling hours: 94.5 h during daytime and 31.5 h during night-time. In each survey, the study sites were thoroughly searched in a zigzag fashion and carefully checked for herpetofauna, including underneath loose objects (e.g. dead wood, bark, leaf litter). We noted the date and time at the beginning of each survey. For each encounter (i.e. observed individual), species and genus were registered. At times, photos were used for ID confirmation. On some occasions, no animals were detected at a study site. These zero-encounter surveys, representing 45 of the 168 sampling events, were excluded from the dataset. This exclusion was necessary because the absence of observations cannot be confidently interpreted as true species absence (MacKenzie 2018).

Opportunistic herpetofauna surveys: These complement the standardised surveys and took place throughout the study area, including all records collected outside of the standardised surveys. As such, opportunistic surveys include all amphibians and reptiles (classes Squamata and Testudines) observed while commuting to and between sampling sites and within the accommodation surroundings. Additionally, specimens found by locals, whose identification we were able to confirm (e.g. road kills), were also included as opportunistic records.

Species identification: Herpetofauna was identified visually based on morphological characters. On some occasions deemed needed and safe, animals were caught for identification (e.g. ridge-count for frogs, scale-count for reptiles). Amphibians were identified with the aid of AmphibiaWeb (AmphibiaWeb 2022) and scientific literature (Pickersgill 2007, Auliya et al. 2012). For reptile identification, Reptile Database (Uetz et al. 2024) and the field guides for snakes (Chippaux and Jackson 2019) and for lizards and turtles (Testudines) (Trape et al. (2012)) were used. Due to the lack of conclusive unique morphological characters for some species and several specimens quickly fleeing, conclusive identification to species level was not always possible. Consequently, 412 observations of amphibians in the standardised dataset and 32 observations (27 amphibians and four squamates) in the opportunistic dataset were identified only at the genus level. As the datasets only include specimens identified accurately to genus or species level, one record identified to the family Leptotyphlopidae was excluded.

## Geographic coverage

### Description

The study took place in northern Guinea-Bissau, Oio Province, in the surroundings of Djalicunda.

**Coordinates**:

Standardised surveys: Latitude: between 12°15'29"N and 12°24'50"N; Longitude: between 15°10'12"W and 15°14'17"W.

Opportunistic occurrences: Latitude: between 12°15'29"N and 12°31'19"N; Longitude: between 15°10'8"W and 15°14'17"W.

## Taxonomic coverage

### Description

**Figure 4.**
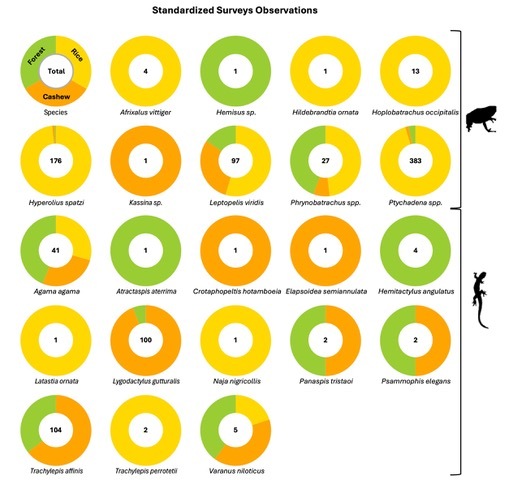
Number of amphibian and reptile records obtained for each species in the standardised surveys in each of the habitat types (i.e. forests, cashew orchards and rice paddies) and corresponding proportion of recordes obtained per habitat-type.

Standardised surveys: This dataset includes a total of 703 amphibian and 265 squamates encounters, corresponding to nine amphibian and 13 squamate taxa (Table 1; Fig. 4; dos Reis-Silva et al. (2024b)).

Opportunistic surveys: This dataset includes 62 amphibian, three testudines and 90 squamates encounters, corresponding to 10 amphibian taxa, two testudine taxa and 25 squamate taxa (Table 2; dos Reis-Silva et al. (2024a)).

### Taxa included

**Table:** 

Rank	Scientific Name	
species	*Afrixalusvittiger* (Peters, 1876)	
species	*Agamaagama* (Linnaeus, 1758)	
species	*Atractaspisaterrima* Günther, 1863	
species	*Bitisarietans* Merrem, 1820	
species	*Boaedonlineatus* Duméril, Bibron & Duméril, 1854	
species	*Caususmaculatus* (Hallowell, 1842)	
species	*Chamaeleogracillis* Hallowell, 1844	
species	*Crotaphopeltishotamboeia* (Laurenti, 1768)	
species	*Dasypeltisconfusa* Trape & Mané, 2006	
species	*Dasypeltis* sp.	
species	*Dendroaspispolylepis* Günther, 1864	
species	*Elapsoideasemiannulata* Bocage, 1882	
species	*Hemidactylusangulatus* Hallowell, 1854	
species	*Hemisus* sp.	
species	*Hildebrandtiaornata* (Peters, 1878)	
species	*Hoplobatrachusoccipitalis* (Günther, 1858)	
species	*Hyperoliusspatzi* Ahl, 1931	
species	*Kassina* sp.	
species	*Kinixysbelliana* Gray, 1831	
species	*Latastiaornata* Monard, 1940	
species	*Leptopelisviridis* (Günther, 1869)	
species	*Lycophidionalbomaculatum* Steindachner, 1870	
species	*Lygodactylusgutturalis* (Bocage, 1873)	
species	*Najanigricollis* Reinhardt, 1843	
species	*Panaspistristaoi* (Monard, 1940)	
species	*Pelusioscastaneus* (Schweigger, 1812)	
species	*Phrynobatrachus* sp.	
species	*Prosymnameleagris* (Reinhardt, 1843)	
species	*Psammophiselegans* (Shaw, 1802)	
species	*Psammophis* sp.	
species	*Ptychadena* sp.	
species	*Pythonregius* (Shaw, 1802)	
species	*Sclerophrysregularis* (Reuss, 1833)	
species	*Sclerophrys* sp.	
species	*Tarentolasenegambiae* Joger, 1984	
species	*Trachylepisaffinis* (Gray, 1838)	
species	*Trachylepiskeroanensis* (Chabanaud, 1921)	
species	*Trachylepisperrotetii* (Duméril & Bibron, 1839)	
species	*Varanusniloticus* (Linnaeus, 1766)	

## Temporal coverage

**Data range:** 2022-6-15 – 2022-11-06.

### Notes

Standardised survey: 18-06-2022 to 05-11-2022; Opportunistic survey: 15-06-2022 to 06-11-2022.

## Usage licence

### Usage licence

Other

### IP rights notes

This work is licensed under a Creative Commons Attribution (CC-BY) 4.0 License.

## Data resources

### Data package title

Amphibian and reptile dataset across different land-use types in Guinea-Bissau, West Africa

### Resource link

https://doi.org/10.15468/vv9xnb; https://doi.org/10.15468/dwectn

### Number of data sets

2

### Data set 1.

#### Data set name

Standardised survey dataset of amphibian and reptile across different land-use types in Guinea-Bissau, West Africa

#### Data format

Darwin Core Archive format

#### Character set

UTF-8

#### Download URL

http://ipt.gbif.pt/ipt/archive.do?r=gw_herpetol_dataset

#### Description

A comprehensive dataset of standardised surveys of amphibians and reptiles (Testudines and Squamata), conducted primarily across forest fragments, cashew orchards and rice paddies in northern Guinea-Bissau, is presented. Standardised surveys were conducted at 21 sampling sites, with seven sites in each habitat type. A total of 703 amphibian and 265 reptile encounters were recorded, corresponding to nine and 13 taxa, respectively (Table 1).

**Data set 1.** 

Column label	Column description
eventID (Event core, Occurrence extention)	An identifier for the set of information associated with a dwc:Event (something that occurs at a place and time).
samplingProtocol (Event core)	The names of, references to, or descriptions of the methods or protocols used during a dwc:Event.
samplingEffort (Event core)	The amount of effort expended during a dwc:Event.
sampleSizeValue (Event core)	A numeric value for a measurement of the size (time duration, length, area or volume) of a sample in a sampling dwc:Event.
sampleSizeUnit (Event core)	The unit of measurement of the size (time duration, length, area or volume) of a sample in a sampling dwc:Event.
habitat (Event core)	A category or description of the habitat in which the dwc:Event occurred.
eventDate (Event core)	The date-time or interval during which a dwc:Event occurred.
eventTime (Event core)	The time or interval during which a dwc:Event occurred.
country (Event core)	The name of the country or major administrative unit in which the dcterms:Location occurs.
countryCode (Event core)	The standard code for the country in which the dcterms:Location occurs.
decimalLatitude (Event core)	The geographic latitude (in decimal degrees, using the spatial reference system given in dwc:geodeticDatum) of the geographic centre of a dcterms:Location.
decimalLongitude (Event core)	The geographic longitude (in decimal degrees, using the spatial reference system given in dwc:geodeticDatum) of the geographic centre of a dcterms:Location.
coordinateUncertaintyInMetres (Event core)	The horizontal distance (in metres) from the given dwc:decimalLatitude and dwc:decimalLongitude describing the smallest circle containing the whole of the dcterms:Location.
geodeticDatum (Event core)	The ellipsoid, geodetic datum or spatial reference system (SRS) upon which the geographic coordinates given in dwc:decimalLatitude and dwc:decimalLongitude are based.
ownerInstitutionCode (Event core)	The name (or acronym) in use by the institution having ownership of the object(s) or information referred to in the record.
institutionID (Event core)	An identifier for the institution having custody of the object(s) or information referred to in the record.
institutionCode (Event core)	The name (or acronym) in use by the institution having custody of the object(s) or information referred to in the record.
basisOfRecord (Occurrence extension)	The specific nature of the data record.
individualCount (Occurrence extension)	The number of individuals present at the time of the dwc:Occurrence.
organismQuantity (Occurrence extension)	A number or enumeration value for the quantity of dwc:Organisms.
organismQuantityType (Occurrence extension)	The type of quantification system used for the quantity of dwc:Organisms.
occurrenceStatus (Occurrence extension)	A statement about the presence or absence of a dwc:Taxon at a dcterms:Location.
scientificName (Occurrence extension)	The full scientific name, with authorship and date information, if known. When forming part of a dwc:Identification, this should be the name in lowest level taxonomic rank that can be determined.
kingdom (Occurrence extension)	The full scientific name of the kingdom in which the dwc:Taxon is classified.
phylum (Occurrence extension)	The full scientific name of the phylum or division in which the dwc:Taxon is classified.
class (Occurrence extension)	The full scientific name of the class in which the dwc:Taxon is classified.
order (Occurrence extension)	The full scientific name of the order in which the dwc:Taxon is classified.
family (Occurrence extension)	The full scientific name of the family in which the dwc:Taxon is classified.
genus (Occurrence extension)	The full scientific name of the genus in which the dwc:Taxon is classified.
specificEpithet (Occurrence extension)	The name of the first or species epithet of the dwc:scientificName.
taxonRank (Occurrence extension)	The taxonomic rank of the most specific name in the dwc:scientificName.
recordedBy (Occurrence extension)	A person, group or organisation responsible for recording the original dwc:Occurrence.
parentEventID (event core)	An identifier for the broader dwc:Event that groups this and potentially other dwc:Events. In this case, a broader category for a sampling site.
occurrenceID (Occurrence extension)	An identifier for the dwc:Occurrence (as opposed to a particular digital record of the dwc:Occurrence).

### Data set 2.

#### Data set name

Opportunistic records of amphibian and reptile across different land-use types in Guinea-Bissau, West Africa

#### Data format

Darwin Core Archive format

#### Character set

UTF-8

#### Download URL

http://ipt.gbif.pt/ipt/archive.do?r=gw_herpetol_occurr_dataset

#### Description

A comprehensive dataset of opportunistic surveys of amphibians and reptiles conducted in northern Guinea-Bissau, Oio Province, in the surroundings of Djalicunda. Opportunistic surveys yielded 62 amphibian, three testudines and 90 squamates encounters, corresponding to 10 amphibian taxa, two testudine taxa and 25 squamate taxa.

**Data set 2.** 

Column label	Column description
occurrenceID	An identifier for the dwc:Occurrence (as opposed to a particular digital record of the dwc:Occurrence).
basisOfRecord	The specific nature of the data record.
eventDate	The date-time when the dwc:Event was recorded.
eventTime	The time or interval during which a dwc:Event occurred.
scientificName	The full scientific name, with authorship and date information, if known. When forming part of a dwc:Identification, this should be the name in lowest level taxonomic rank that can be determined.
kingdom	The full scientific name of the kingdom in which the dwc:Taxon is classified.
phylum	The full scientific name of the phylum or division in which the dwc:Taxon is classified.
class	The full scientific name of the class in which the dwc:Taxon is classified.
order	The full scientific name of the order in which the dwc:Taxon is classified.
family	The full scientific name of the family in which the dwc:Taxon is classified.
genus	The full scientific name of the genus in which the dwc:Taxon is classified.
specificEpithet	The name of the first or species epithet of the dwc:scientificName.
taxonRank	The taxonomic rank of the most specific name in the dwc:scientificName.
lifeStage	The age class or life stage of the dwc:Organism(s) at the time the dwc:Occurrence was recorded.
decimalLatitude	The geographic latitude (in decimal degrees, using the spatial reference system given in dwc:geodeticDatum) of the geographic centre of a dcterms:Location.
decimalLongitude	The geographic longitude (in decimal degrees, using the spatial reference system given in dwc:geodeticDatum) of the geographic centre of a dcterms:Location.
coordinateUncertaintyInMetres	The horizontal distance (in metres) from the given dwc:decimalLatitude and dwc:decimalLongitude describing the smallest circle containing the whole of the dcterms:Location.
geodeticDatum	The ellipsoid, geodetic datum or spatial reference system (SRS) upon which the geographic coordinates given in dwc:decimalLatitude and dwc:decimalLongitude are based.
country	he name of the country or major administrative unit in which the dcterms:Location occurs.
countryCode	The standard code for the country in which the dcterms:Location occurs.
institutionID	An identifier for the institution having custody of the object(s) or information referred to in the record.
institutionCode	The name (or acronym) in use by the institution having custody of the object(s) or information referred to in the record.
recordedBy	A list (concatenated and separated) of names of people, groups or organisations responsible for recording the original dwc:Occurrence.
individualCount	The number of individuals present at the time of the dwc:Occurrence.
organismQuantity	A number or enumeration value for the quantity of dwc:Organisms.
organismQuantityType	The type of quantification system used for the quantity of dwc:Organisms.

## Figures and Tables

**Table 1.** Amphibian and reptile (Squamata) observations during standardised surveys in northern Guinea-Bissau, West Africa. For each species, we provide the scientific name, as well as common name and IUCN status (IUCN 2025) whenever available. We also provide the number of observations for each species, including the proportion of records calculated considering amphibians (Amphibia) and 2) reptiles (Squamata) separately.

**Class**	**Family**	**Species (IUCN status)**	**Number of observations (%)**
Amphibia	Arthroleptidae	*Leptopelisviridis* (Günther, 1869), (LC)	97 (13.8%)
	Hyperoliidae	*Afrixalusvittiger* (Peters, 1876), Savanna Banana Frog (LC)	4 (0.6%)
	Hyperoliidae	*Hyperoliusspatzi* (Ahl, 1931), (LC)	176 (25.0%)
	Hyperoliidae	*Kassina* sp.	1 (0.1%)
	Ptychadenidae	*Ptychadena* sp.	383 (54.5%)
	Ptychadenidae	*Hildebrandtiaornata* (Peters, 1878), African Ornate Frog (LC)	1 (0.1%)
	Hemisotidae	*Hemisus* sp.	1 (0.1%)
	Dicroglossidae	*Hoplobatrachusoccipitalis* (Günther, 1858), African Groove-crowned Frog (LC)	13 (1.8%)
	Phrynobatrachidae	*Phrynobatrachus* sp.	27 (3.8%)
Squamata	Varanidae	*Varanusniloticus* (Linnaeus, 1766), Nile Monitor (LC)	5 (1.9%)
	Agamidae	*Agamaagama* (Linnaeus, 1758), Common Agama (LC)	41 (15.5%)
	Gekkonidae	*Hemidactylusangulatus* (Hallowell, 1854), House Gecko (LC)	4 (1.5%)
	Gekkonidae	*Lygodactylusgutturalis* (Bocage, 1873), Chevron-throated Dwarf Gecko (LC)	100 (37.8%)
	Scincidae	*Trachylepisaffinis* (Gray, 1838), Senegal Mabuya (LC)	104 (30.4%)
	Scincidae	*Trachylepisperrotetii* (Duméril & Bibron, 1839), Teita Mabuya (LC)	2 (0.8%)
	Scincidae	*Panaspistristaoi* (Monard, 1940), Tristoi's Snake-eyed Skink (LC)	2 (0.8%)
	Lacertidae	*Latastiaornata* (Monard, 1940), (DD)	1 (0.4%)
	Lamprophiidae	*Atractaspisaterrima* (Günther, 1863), Mole Viper (LC)	1 (0.4%)
	Lamprophiidae	*Psammophiselegans* (Shaw, 1802), Elegant Sand Racer (LC)	2 (0.8%)
	Colubridae	*Crotaphopeltishotamboeia* (Laurenti, 1768), Red-lipped Snake (LC)	1 (0.4%)
	Elapidae	*Elapsoideasemiannulata* (Bocage, 1882), Angolan Garter Snake (LC)	1 (0.4%)
	Elapidae	*Najanigricollis* (Reinhardt, 1843), Black-necked Spitting Cobra (LC)	1 (0.4%)

**Table 2.** List of amphibian and reptile species (classes Testudines and Squamata) opportunistically detected in Guinea-Bissau, West Africa. For each species, we provide the scientific name and, whenever available, the common name and the IUCN status (IUCN 2025). The number of observations is also provided for each species, as well as the corresponding proportions calculated considering amphibians (Amphibia) and reptiles (Testudines + Squamata) separately.

**Class**	**Family**	**Species (IUCN status)**	**Number of observations (%)**
Amphibia	Arthroleptidae	*Leptopelisviridis* (Günther, 1869) (LC)	15 (24.2%)
	Hyperoliidae	*Afrixalusvittiger* (Peters, 1876), Savanna Banana Frog (LC)	4 (6.5%)
	Hyperoliidae	*Hyperoliusspatzi* (Ahl, 1931) (LC)	8 (12.9%)
	Ptychadenidae	*Ptychadena* sp.	9 (14.1%)
	Ptychadenidae	*Hildebrandtiaornata* (Peters, 1878), African Ornate Frog (LC)	1 (1.6%)
	Hemisotidae	*Hemisus* sp.	2 (3.1%)
	Dicroglossidae	*Hoplobatrachusoccipitalis* (Günther, 1858), African Groove-crowned Frog (LC)	6 (9.4%)
	Phrynobatrachidae	*Phrynobatrachus* sp.	3 (4.7%)
	Bufonidae	*Sclerophrys* sp.	13 (20.3%)
	Bufonidae	*Sclerophrysregularis* (Reuss, 1833) (LC)	1 (1.6%)
Testudines	Testudinidae	*Kinixysbelliana* (Gray, 1831)	2 (2.2%)
	Pelomedusidae	*Pelusioscastaneus* (Schweigger, 1812)	1 (1.1%)
Squamata	Varanidae	*Varanusniloticus* (Linnaeus, 1766), Nile Monitor (LC)	16 (17.2%)
	Agamidae	*Agamaagama* (Linnaeus, 1758), Common Agama (LC)	7 (7.5%)
	Chamaeleonidae	*Chamaeleogracillis* (Hallowell, 1844), Slender Chameleon (LC)	11 (11.8%)
	Gekkonidae	*Hemidactylusangulatus* (Hallowell, 1854), House Gecko (LC)	7 (7.5%)
	Gekkonidae	*Lygodactylusgutturalis* (Bocage, 1873), Chevron-throated Dwarf Gecko (LC)	5 (5.4%)
	Phyllodactylidae	*Tarentolasenegambiae* (Joger, 1984) (LC)	2 (2.2%)
		*Trachylepisaffinis* (Gray, 1838), Senegal Mabuya (LC)	2 (2.2%)
		*Trachylepiskeroanensis* (Chabanaud, 1921), (DD)	3 (3.2%)
	Scincidae	*Trachylepisperrotetii* (Duméril & Bibron, 1839), Teita Mabuya (LC)	5 (5.4%)
		*Panaspistristaoi* (Monard, 1940), Tristoi's Snake-eyed Skink (LC)	2 (2.2%)
		*Latastiaornata* (Monard, 1940) (DD)	4 (4.3%)
	Lamprophiidae	*Boaedonlineatus* (Duméril, Bibron & Duméril, 1854), Striped House Snake (LC)	2 (2.2%)
		*Lycophidionalbomaculatum* (Steindachner, 1870), (LC)	3 (3.2%)
	Lamprophiidae	*Psammophis* sp.	4 (4.3%)
	Lamprophiidae	*Psammophiselegans* (Shaw, 1802), Elegant Sand Racer (LC)	1 (1.1%)
	Prosymnidae	*Prosymnameleagris* (Reinhardt, 1843), Ghana Shovel-snout (LC)	3 (3.2%)
	Colubridae	*Crotaphopeltishotamboeia* (Laurenti, 1768), Red-lipped Snake (LC)	2 (2.2%)
		*Dasypeltis* sp. (LC)	1 (1.1%)
	Colubridae	*Dasypeltisconfusa* (Trape & Mané, 2006), Diamond-back Egg-eater (LC)	1 (1.1%)
	Pythonidae	*Pythonregius* (Shaw, 1802), Ball Python (NT)	2 (2.2%)
		*Dendroaspispolylepis* (Günther, 1864), Black Mamba (LC)	1 (1.1%)
	Elapidae	*Elapsoideasemiannulata* (Bocage, 1882), Angolan Garter Snake (LC)	2 (2.2%)
		*Najanigricollis* (Reinhardt, 1843), Black-necked Spitting Cobra (LC)	1 (1.1%)
	Viperidae	*Bitisarietans* (Merrem, 1820), Puff Adder (LC)	2 (2.2%)
		*Caususmaculatus* (Hallowell, 1842), Spotted Night Adder (LC)	1 (1.1%)

## References

[B12269415] AmphibiaWeb (2022). https://amphibiaweb.org. https://amphibiaweb.org.

[B12269070] Auliya Mark, Böhme Wolfgang, Wagner Philipp (2012). The herpetofauna of the Bijagós archipelago, Guinea-Bissau (West Africa) and a first country-wide checklist. Bonn Zoological Bulletin.

[B12269079] Catarino L., Martins E. S., Basto M. F. Pinto, Diniz M. A. (2008). An annotated checklist of the vascular flora of Guinea-Bissau (West Africa). Blumea - Biodiversity, Evolution and Biogeography of Plants.

[B12269088] Catarino Luís, Menezes Yusufo, Sardinha Raul (2015). Cashew cultivation in Guinea-Bissau – risks and challenges of the success of a cash crop. Scientia Agricola.

[B12685039] Chaves Patrícia, Schaafsma Marije, Dabo Djunco, Lomba Judite, Mane Fode, de Lima Ricardo, Palmeirim Jorge, Rocha Ricardo, Seck Sambu, Biai Justino, Timóteo Sérgio, Meyer Christoph, Rainho Ana (2024). Friend or foe? Attitudes of rice farmers toward wild animals in West Africa. Ecology and Society.

[B12269573] Chippaux Jean-Philippe, Jackson Kate (2019). Snakes of Central and Western Africa.

[B12269097] Cox Neil, Young Bruce E., Bowles Philip, Fernandez Miguel, Marin Julie, Rapacciuolo Giovanni, Böhm Monika, Brooks Thomas M., Hedges S. Blair, Hilton-Taylor Craig, Hoffmann Michael, Jenkins Richard K. B., Tognelli Marcelo F., Alexander Graham J., Allison Allen, Ananjeva Natalia B., Auliya Mark, Avila Luciano Javier, Chapple David G., Cisneros-Heredia Diego F., Cogger Harold G., Colli Guarino R., De Silva Anslem, Eisemberg Carla C., Els Johannes, Fong G. Ansel, Grant Tandora D., Hitchmough Rodney A., Iskandar Djoko T., Kidera Noriko, Martins Marcio, Meiri Shai, Mitchell Nicola J., Molur Sanjay, Nogueira Cristiano De C., Ortiz Juan Carlos, Penner Johannes, Rhodin Anders G. J., Rivas Gilson A., Rödel Mark-Oliver, Roll Uri, Sanders Kate L., Santos-Barrera Georgina, Shea Glenn M., Spawls Stephen, Stuart Bryan L., Tolley Krystal A., Trape Jean-François, Vidal Marcela A., Wagner Philipp, Wallace Bryan P., Xie Yan (2022). A global reptile assessment highlights shared conservation needs of tetrapods. Nature.

[B12269475] dos Reis-Silva Francisco, Alves-Martins Fernanda, Martinez-Arribas Javier, Pizzigalli Cristian, Seck Sambu, Rainho Ana, Rocha Ricardo, Palmeirim Ana Filipa (2024). Opportunistic records of amphibian and reptile across different land-use types in Guinea-Bissau, West Africa. https://www.gbif.org/dataset/69f9ff24-1e42-462c-9356-a8ec4bda358e.

[B12269487] dos Reis-Silva Francisco, Alves-Martins Fernanda, Martinez-Arribas Javier, Pizzigalli Cristian, Seck Sambu, Rainho Ana, Rocha Ricardo, Palmeirim Ana Filipa (2024). Standardized survey dataset of amphibian and reptile across different land-use types in Guinea-Bissau, West Africa. https://www.gbif.org/dataset/e6db1274-9fca-469f-9f64-f1057555cb2b.

[B12457241] Dos Reis‐Silva Francisco, Pizzigalli Cristian, Seck Sambu, Cabeza Mar, Rainho Ana, Rocha Ricardo, Palmeirim Ana Filipa (2025). Unveiling how herpetofauna cope with land‐use changes—Insights from forest‐cashew‐rice landscapes in West Africa. Biotropica.

[B12269458] FAO (2021). Food systems evaluation discusses food security needs in Guinea-Bissau. https://www.fao.org/countryprofiles/news-archive/detail-news/en/c/1471318/.

[B12269169] Gardner Toby A., Barlow Jos, Chazdon Robin, Ewers Robert M., Harvey Celia A., Peres Carlos A., Sodhi Navjot S. (2009). Prospects for tropical forest biodiversity in a human‐modified world. Ecology Letters.

[B12269181] Gibson Luke, Lee Tien Ming, Koh Lian Pin, Brook Barry W., Gardner Toby A., Barlow Jos, Peres Carlos A., Bradshaw Corey J. A., Laurance William F., Lovejoy Thomas E., Sodhi Navjot S. (2011). Primary forests are irreplaceable for sustaining tropical biodiversity. Nature.

[B12269197] Guedes Jhonny J. M., Moura Mario R., Alexandre F. Diniz‐Filho José (2023). Species out of sight: elucidating the determinants of research effort in global reptiles. Ecography.

[B12685120] Guedes Patrícia, Palmeirim Ana Filipa, Monteiro Filipa, Catarino Luís, Palma Luís, Temudo Marina P., Henriques Mohamed, Beja Pedro, Lopes Ricardo Jorge, Ladle Richard J., Powell Luke L. (2024). At the tipping point: Can biodiversity and rural livelihoods endure uncontrolled cashew expansion in West Africa?. Biotropica.

[B12269206] Hortal Joaquín, De Bello Francesco, Diniz-Filho José Alexandre F., Lewinsohn Thomas M., Lobo Jorge M., Ladle Richard J. (2015). Seven shortfalls that beset large-scale knowledge of biodiversity. Annual Review of Ecology, Evolution, and Systematics.

[B12689532] IUCN The IUCN Red List of Threatened Species. Version 2024-2.. https://www.iucnredlist.org.

[B12685111] Jenkins Clinton N., Pimm Stuart L., Joppa Lucas N. (2013). Global patterns of terrestrial vertebrate diversity and conservation. Proceedings of the National Academy of Sciences.

[B12269217] Lewin Amir, Feldman Anat, Bauer Aaron M., Belmaker Jonathan, Broadley Donald G., Chirio Laurent, Itescu Yuval, LeBreton Matthew, Maza Erez, Meirte Danny, Nagy Zoltán T., Novosolov Maria, Roll Uri, Tallowin Oliver, Trape Jean‐François, Vidan Enav, Meiri Shai (2016). Patterns of species richness, endemism and environmental gradients of African reptiles. Journal of Biogeography.

[B12269528] MacKenzie Darryl I. (2018). Occupancy estimation and modeling: inferring patterns and dynamics of species occurrence.

[B12269239] Meiri Shai, Bauer Aaron M., Allison Allen, Castro‐Herrera Fernando, Chirio Laurent, Colli Guarino, Das Indraneil, Doan Tiffany M., Glaw Frank, Grismer Lee L., Hoogmoed Marinus, Kraus Fred, LeBreton Matthew, Meirte Danny, Nagy Zoltán T., Nogueira Cristiano De C., Oliver Paul, Pauwels Olivier S. G., Pincheira‐Donoso Daniel, Shea Glenn, Sindaco Roberto, Tallowin Oliver J. S., Torres‐Carvajal Omar, Trape Jean‐Francois, Uetz Peter, Wagner Philipp, Wang Yuezhao, Ziegler Thomas, Roll Uri (2018). Extinct, obscure or imaginary: The lizard species with the smallest ranges. Diversity and Distributions.

[B12269273] Myers Norman, Mittermeier Russell A., Mittermeier Cristina G., Da Fonseca Gustavo A. B., Kent Jennifer (2000). Biodiversity hotspots for conservation priorities. Nature.

[B12269283] Newbold Tim, Oppenheimer Philippa, Etard Adrienne, Williams Jessica J. (2020). Tropical and Mediterranean biodiversity is disproportionately sensitive to land-use and climate change. Nature Ecology & Evolution.

[B12269325] Pauwels Olivier S. G., Das Sunandan, Camara Lewei Boyo, Chirio Laurent, Doumbia Joseph, D’Acoz Cédric D’Udekem, Dufour Sylvain, Margraf Nicolas, Sonet Gontran (2023). Rediscovery, range extension, phylogenetic relationships and updated diagnosis of the Ornate Long-tailed Lizard *Latastiaornata* Monard, 1940 (Squamata: Lacertidae). Zootaxa.

[B12269316] Pickersgill Martin (2007). A redefinition of *Afrixalusfulvovittatus* (Cope, 1860) and *Afrixalusvittiger* (Peters, 1876) (Amphibia, Anura
Hyperoliidae). African Journal of Herpetology.

[B12269339] Powers Ryan P., Jetz Walter (2019). Global habitat loss and extinction risk of terrestrial vertebrates under future land-use-change scenarios. Nature Climate Change.

[B12269348] Rege Anushka, Lee Janice Ser Huay (2023). The socio-environmental impacts of tropical crop expansion on a global scale: A case study in cashew. Biological Conservation.

[B12688845] Rossinyol-Fernàndez Aina, Dabo Djunco, dos Reis Silva Francisco, Oliveira Raquel, Seck Sambú, Rainho Ana, Cabeza Mar, Palmeirim Ana Filipa (2024). Use of native and human-modified habitats by different mammal guilds in West Africa. Global Ecology and Conservation.

[B12269357] Sottomayor Madalena, Palmeirim Ana Filipa, Meyer Christoph F. J., De Lima Ricardo F., Rocha Ricardo, Rainho Ana (2024). Nature-based solutions to increase rice yield: An experimental assessment of the role of birds and bats as agricultural pest suppressors in West Africa. Agriculture, Ecosystems & Environment.

[B12269377] Temudo Marina Padrão, Abrantes Manuel Bivar (2013). Changing Policies, shifting livelihoods: The fate of agriculture in Guinea‐Bissau. Journal of Agrarian Change.

[B12269386] Temudo Marina Padrão, Abrantes Manuel (2014). The cashew frontier in Guinea-Bissau, West Africa: Changing landscapes and livelihoods. Human Ecology.

[B12269395] Trape Jean-François, Trape Sébastien, Chirio Laurent (2012). Lézards, crocodiles et tortues d’Afrique occidentale et du Sahara.

[B12269499] Uetz Peter, Freed P., Aguilar R., Reyes F., Kudera J., Hošek J. (2024). The reptile database. http://www.reptile-database.org.

[B12685136] Vasconcelos Sasha, Rodrigues Patrícia, Palma Luís, Mendes Luís F., Palminha Agostinho, Catarino Luís, Beja Pedro (2015). Through the eye of a butterfly: Assessing biodiversity impacts of cashew expansion in West Africa. Biological Conservation.

[B12269509] WHO (2023). Snakebite envenoming. https://www.who.int/news-room/fact-sheets/detail/snakebite-envenoming.

